# Development of Crystalline Morphology and Its Relationship with Mechanical Properties of PP/PET Microfibrillar Composites Containing POE and POE-*g*-MA

**DOI:** 10.3390/polym10030291

**Published:** 2018-03-08

**Authors:** Maja Kuzmanović, Laurens Delva, Dashan Mi, Carla Isabel Martins, Ludwig Cardon, Kim Ragaert

**Affiliations:** 1 Centre for Polymer and Material Technologies, Department of Materials, Textiles and Chemical Engineering, Faculty of Engineering and Architecture, Ghent University, Technologiepark 915, Zwijnaarde 9052, Belgium; maja.kuzmanovic@ugent.be (M.K.); laurens.delva@ugent.be (L.D.); dashan.mi@ugent.be (D.M.); ludwig.cardon@ugent.be (L.C.); 2 College of Polymer Science and Engineering, State Key Laboratory of Polymer Materials Engineering, Sichuan University, Chengdu 610065, China; 3 Institute for Polymer and Composites/I3N, University of Minho, Campus de Azurém, 4800-058 Guimarães, Portugal; cmartins@dep.uminho.pt

**Keywords:** microfibrillar composites, crystalline morphology, crystallinity, mechanical properties

## Abstract

The main goal of this research is to study the development of crystalline morphology and compare it to various mechanical properties of microfibrillar composites (MFCs) based on polypropylene (PP) and poly(ethylene terephthalate) (PET), by adding a functional compatibilizer and a non-functional rubber in two different steps in the processing sequence. The MFCs were prepared at a weight ratio of 80/20 PP/PET by twin screw extrusion followed by cold drawing and injection moulding. The non-functionalized polyolefin-based elastomer (POE) and the functional compatibilizer (i.e., POE grafted with maleic anhydride (POE-*g*-MA)) were added in a fixed weight percentage at two stages: during extrusion or during injection moulding. The morphology observations showed differences in crystalline structure, and the PP spherulite size was reduced in all MFCs due to the presence of PET fibrils. Their relationship with the mechanical performances of the composite was studied by tensile and impact tests. Adding the functional compatibilizer during extrusions showed better mechanical properties compared to MFCs. Overall, a clear relationship was identified between processing, structure and properties.

## 1. Introduction

Fibre reinforced composites have attracted great attention in the last decades. It is well known that incorporating different inorganic fibres into polymer matrices such as glass fibres or carbon fibres significantly improves mechanical performance, by enhancing strength and stiffness [1,2,3]. However, these types of fibre composites are not very environment-friendly, as they are difficult to mechanically recycle because of issues in the separation of the different components. This may be avoided with polymer-polymer reinforced composites, which can easily be mechanically recycled. In this respect, microfibrillar composites (MFCs) could be interesting as their improved mechanical properties allow them to be used in a wide range of applications. The MFC concept is a methodology developed in the early nineties by Fakirov and Evstatiev [4]. MFCs are a type of polymer-polymer composites in which a high-melting fibrillar thermoplastic polymer reinforces a lower-melting one [4]. In our recent study [5], the importance of the processing parameters during the production of MFCs has been pointed out, but besides these parameters, the composition of the starting mixture, the viscosity ratio and the compatibility of the components are equally important [6].

The research on polymer blends and polymer-polymer composites has also led to an increased interest in compatibilization. Numerous studies have been conducted on the compatibilization of blends, using different compatibilizers [7,8,9,10,11] and improving the dispersion of the second phase. The compatibilizer can be concentrated in the interface between two polymers during blending, thus preventing coalescence and resulting in smaller and finer dispersions as well as better adhesion between the phases [12,13,14].

**Figure 1:**
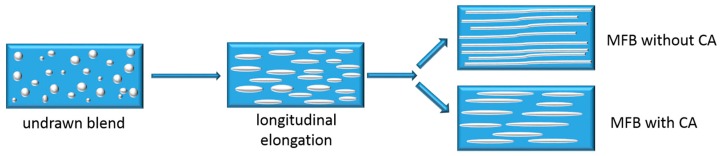
Morphology of blend during cold drawing with and without compatibilizer added during extrusion [4].

On the other hand, it is also known that the MFC concept relies on the incompatibility between the matrix and dispersed phase, and that the final mechanical properties depend on the aspect ratio of the reinforcement and the interfacial adhesion between the matrix and reinforcement [4]. Various authors [4,15] have proposed that combining these two approaches, the MFC concept and compatibilization, could improve the properties of the final composites. According to Fakirov et al. [4], MFCs without compatibilizer can reach fibril lengths of up to 200 µm because the coalescence will take place during blending and drawing. In cases with a compatibilizer, Friedrich et al. [16] found a decrease in the tensile modulus and strength for the PP/PET in situ compatibilized microfibrillar blends. They attributed this reduction to the shorter microfibrils caused by the use of a compatibilizer, which covers the PET particles during melt blending and prevents their coalescence during drawing. This change in morphology can be seen in Figure 1. Fakirov et al. [15] reported in one of their studies that a compatibilizer affects the length of the fibrils depending on in which step of the processing sequence it is added. They have therefore suggested adding a compatibilizer to the drawn blend, in the final processing step, in injection or compression moulding. At this stage, the compatibilizer should facilitate distribution of the fibrils, improve the interfacial adhesion between matrix and fibrils, and enhance mechanical properties without reducing the aspect ratio of the fibrils. However, there are no experimental results to support this theory. To fill this gap, the present study investigates the effect of adding a compatibilizer during both extrusion and injection moulding. To this end, we have selected both a non-functionalized rubber and a functional compatibilizer, as a difference in migration to the interface and reactivity is expected.

To reveal the origin of ductile or brittle behaviour, researchers typically focus on the influence of crystallinity and crystalline structure of the composites. Embrittlement is known to be the result of the high crystallinity of semicrystalline polymers, but the size and perfection of the spherulites also play an important role in this behaviour [17,18]. In the case of semicrystalline polymers and their composites, different processing conditions can affect the crystalline structure, such as the perfection of crystallites, spherulite growth and orientation of the lamellae [19]. The process of making the MFCs may cause changes in the crystalline morphology of the matrix. The fibres could act as heterogeneous nucleating agents for the matrix, in which these nuclei can induce the crystal growth in the lateral direction [2,20,21].

To shed more light on this, the aim of this study is to examine the relationships between the development of the microstructure, the crystallinity and the mechanical properties of the PP/PET microfibrillar composites (MFCs). A polyolefin-based elastomer (POE) and POE grafted with maleic anhydride (POE-*g*-MA) will be added in a fixed weight percentage during extrusion and injection moulding, and we will investigate how this affects crystalline morphology and properties.

This research study will provide better insight into the morphological and crystallinity development of MFCs during processing. An alternative approach will be suggested to achieve good mechanical properties of MFCs.

## 2. Materials and Methods

### 2.1. Materials

Polypropylene (PP) was purchased from Sabic (Sabic 575P, Bergen op Zoom, The Netherlands) with a melt flow rate (MFR) of 11 g/10 min (2.16 kg, 230 °C), and the used PET was LIGHTER C93 from Equipolymers (Schkopau, Germany), which is a bottle-grade material with an intrinsic viscosity of 0.80 ± 0.02 dL/g. PET was dried in a vacuum oven for 15 h at 80 °C, and 2 h before processing at 120 °C, while PP was used as received. As additives, rubber and a compatibilizer were used in this research. POE (Vistamaxx^TM^ 6102), which is an ethylene-propylene elastomer with a MFR of 3 g/10 min (2.16 kg, 230 °C), was kindly provided by ExxonMobil (Machelen, Belgium). POE-*g*-MA used in this study was Acti-Tech 16MA13, which is a Vistamaxx-based compatibilizer, kindly donated by Nordic Grafting Company (NGC, Hellerup, Denmark). The grafting percentage of the MA group onto the backbone of the compatibilizer was 1.3 wt %, according to the data sheet. Both rubber and compatibilizer were dried at 60 °C for 15 h before processing.

### 2.2. Preparation of MFCs

In this study, PP was used as a matrix and PET as a reinforcing element. The samples were prepared in a weight ratio of 80/20 PP/PET and POE or POE-*g*-MA were added in 6 wt %, while the same PP/PET ratio was maintained. The PP/PET weight ratio was determined based on previous proprietary research. The MFCs were prepared by extrusion followed by cold drawing and injection moulding. Five different samples were prepared: non-compatibilized MFC, MFCs with POE and POE-*g*-MA added in the extrusion step (POE_EXT_ and POE-*g*-MA_EXT_), and MFCs with POE and POE-*g*-MA added in the injection moulding step (POE_IM_ and POE-*g*-MA_IM_) (Table 1).

The melt blending of polymers with and without additive was conducted with a twin-screw extruder (Coperion ZSK18, Stuttgart, Germany) with two co-rotating screws of 18 mm diameter, *L*/*D* = 40 and a die opening of 19 mm × 2 mm. The screw speed was set at 120 rpm and the barrel temperatures were set between 205 and 260 °C. The extrudate was obtained as a sheet with dimensions of 25 mm × 1 mm, by passing through calender rolls, which were cooled down to ~15 °C. The received cooled extrudate entered directly into a hot oven (200 °C, 55.5 cm × 60 cm) and cold drawn by a pair of rolls above the glass transition temperature of PET. During drawing, the surface temperature of the extrudate was measured and amounted to approximately 95 °C. The speed of the rolls was adjusted to obtain a draw ratio of 8. Injection moulding was performed on an Engel 80T, with a temperature profile of 180, 190, 200 and 210 °C in a standard mould with a temperature of 30 °C, thus obtaining both tensile (114 × 6.45 × 4 mm^3^, with a gauge length of 33 mm) and impact specimens (126 × 13 × 3 mm^3^).

### 2.3. Characterization

#### 2.3.1. Structural Characterization

Polarized optical microscopy (POM) Leica DM 2500 P (Wetzlar, Germany) was used to study the morphology of the specimens. Thin slices of 15 µm were cut from the injection moulded samples with a microtome Leica RM2245 (Wetzlar, Germany) in the direction parallel to the injection flow. They were subsequently inserted between two microscope cover glasses and glued with Canadian balm. Samples were analysed with a Leica Camera type DFC425 and DFC360FX (Wetzlar, Germany).

To determine the spherulites size, small angle light scattering (SALS) experiments were performed. For this, injection moulded samples were microtomed into 15 µm thick layers parallel to the flow direction on the FD-TD plane [22]. To suppress surface scattering, they were immersed in Canadian balm between two microscopic slides. Next, to obtain the SALS patterns, a 632.8 nm He-Ne laser with beam size of 1 mm was used as the source of polarized monochromatic light. SALS *H*_v_ patterns were captured using a Hamamatsu digital camera (Hamamatsu Photonics K.K., Hamamatsu City, Japan) and analysed with Hipic 6.3.0 software (Hamamatsu Photonics K.K., Hamamatsu City, Japan). The equivalent radius (*R*_0_) of the spherulites was estimated with the following Equation (1) [23]:

(1) $$R_{0}=\frac{1025}{\pi }\frac{\lambda }{\sin\left(\theta_{\max}/2\right)},$$

*λ* is the wavelength of light in the medium. The distance from the centre of the *H*_v_ pattern to the intensity maximum in one lobe, in conjugation with the known sample-to-film distance, is a measure of the polar angle *θ*_max_. (*θ*_max_ = tan (distance from the centre of the *H*_v_ pattern to the intensity maximum in one lobe/sample-to-film distance)).

To study the phase and crystalline morphology of MFCs, we used scanning electron microscopy (SEM) FEG SEM JEOL JSM-7600F 202 (Tokyo, Japan). The samples were immersed in liquid nitrogen and subsequently fractured. For the observation of the phase morphology, the PP matrix together with POE was selectively dissolved in hot xylene for 1–3 h. For the crystalline morphology observation of the composites, the amorphous phase of the PP and PET was chemically etched in a solution H_2_SO_4_–H_3_PO_4_–KMnO_4_ at 70 °C for 5–6 h. Furthermore, the samples surfaces were sputtered with gold by a Bal-Tec SCD005 sputter coater (Bal-Tec, Balzers, Liechtenstein). Micrographs were obtained with an accelerating voltage of 20 kV. The average diameter of the fibrils was calculated with Image J software (National Institute of Health, Bethesda, MD, USA). For the calculation, at least 50 measurements were used.

X-ray diffraction (XRD) measurements were carried out to confirm the crystal modification of PP. Tests were performed on a Bruker D8 Discover XRD system (Bruker Nederland BV, Leiden, The Netherlands) equipped with a Cu X-ray source (λ = 1.5406 Å) and a linear X-ray detector. The samples were put on a Si sample cup on the sample heating stage. θ–2θ measurements were carried out in air at atmospheric pressure at a temperature of 24 °C.

The structure-related thermal properties of the composites were determined via differential scanning calorimetry (DSC). Analysis was performed on a Netzsch DSC 214 Polyma device (Selb, Germany) in one cycle of heating-cooling in the temperature range between 20–200 °C. The tests ran under nitrogen atmosphere; the flow of nitrogen gas was 20 mL·min^−1^ and the heating/cooling rate was 10 °C·min^−1^. Crystallinity (α_c_) was calculated for the PP phase based on the theoretical enthalpy for 100% crystalline polymer and taking the mass percentage into account (Equation (2)):

(2) $$\;\alpha_{c}=\frac{\Delta H^{exp}}{\Delta H^{{}^{\circ}}w_{f}}\;\cdot 100\%,$$

where standard enthalpy (Δ*H*°) for PP is 207 J·g^−1^ [24], and $w_{f}$ is the weight fraction of the relevant polymer in the PP/PET mixture. The mean thermal properties were averaged from three measurements and the differences were calculated by comparing population means by t-independent test via the software package SPSS Statistics 24 (Armonk, NY, USA).

#### 2.3.2. Mechanical Characterization

Mechanical characterization was conducted under controlled conditions (23 °C and 50% relative humidity), after the samples had been conditioned for a minimum of 48 h within this controlled environment. The standard tensile bars were tested with an Instron 5565 tensile device (Norwood, MA, USA) according to standard ISO 527. During the tests, different test speeds were used before and after the Instron dynamic extensometer was removed (type catalogue 2620-603 with a gauge length of 12.5 mm), 1 mm/min and 5 mm/min, respectively. Analysis was performed with Bluehill software.

The notched Charpy impact test was used to evaluate the toughness of the samples by using a Tinius Olsen IT 503 Pendulum Impact Tester (Ulm, Germany) according to ISO 179. The specimens were notched in the middle of the sample with a depth of 2 mm, placed horizontally with the notch oriented away from the pendulum and broken by a hammer with an energy of 2 J. At least five specimens were tested for both tensile and impact tests. The differences between the samples are calculated by t-independent test preceded by a Levene’s test for equality of variance via the software package SPSS Statistics 24 (Armonk, NY, USA) with a probability value of 0.05.

## 3. Results and Discussion

### 3.1. Morphology Development

**Figure 2:**
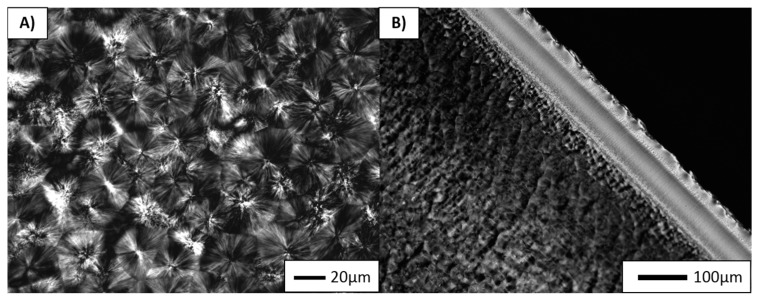
POM micrographs of neat PP sample in transferred direction to the injection flow.

Polarized optical microscopy was found to be a simple method to distinguish the changes in crystalline structure, such as the growth of crystals and their orientation [25]. Micrographs of neat PP sample are represented in Figure 2A,B. As can be seen, the micrographs show a clear spherulitic structure (Figure 2A) of PP, and due to the injection moulding process a typical “skin–core” structure (Figure 2B) can be discerned [19]. The average PP crystal size, measured quantitively by SALS, was found to be 22.3 µm (Table 2).

**Figure 3:**
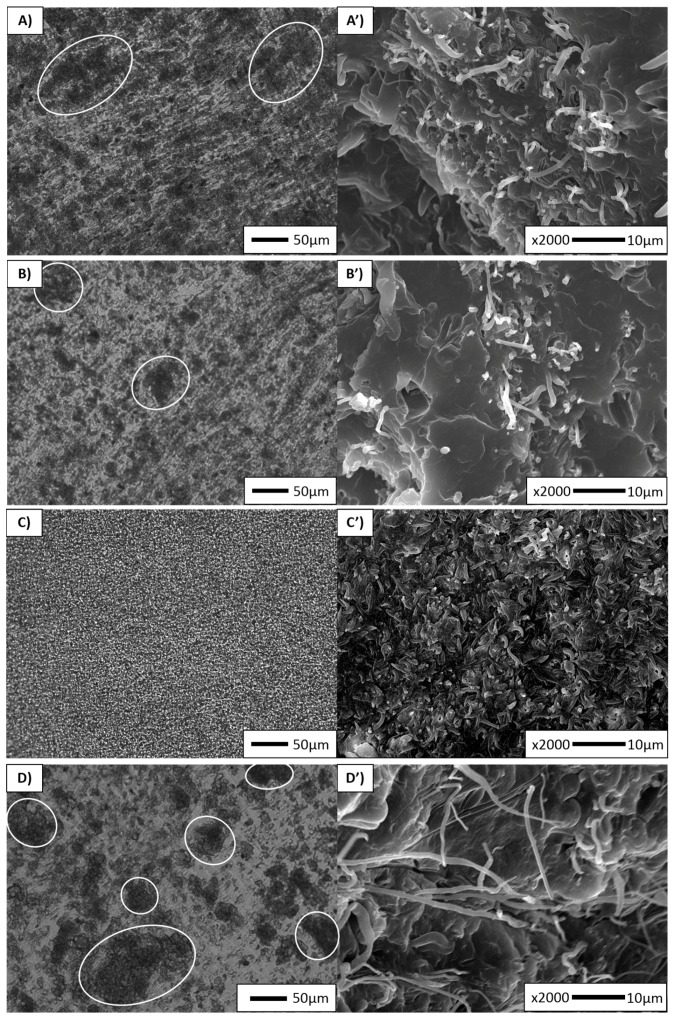

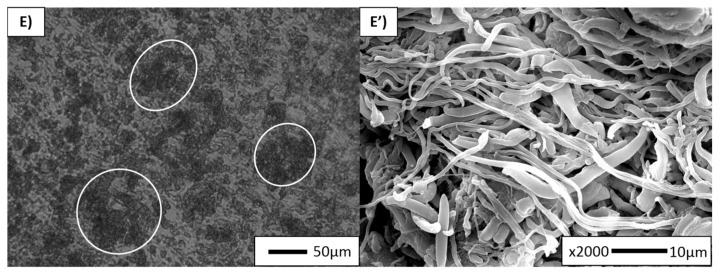
Microstructures of MFCs samples obtained via POM along the flow direction: (**A**) MFC; (**B**) POE_EXT_; (**C**) POE-*g*-MA_EXT_; (**D**) POE_IM_; (**E**) POE-*g*-MA_IM_. Microstructures of MFCs samples obtained via SEM in transverse direction: (**A’**) MFC (PP partially etched after 1.5 h in hot xylene); (**B’**) POE_EXT_ (PP partially etched after 1.5 h in hot xylene); (**C’**) POE-*g*-MA_EXT_ (PP partially etched after 3 h in hot xylene); (**D’**) POE_IM_ (PP partially etched after 3 h in hot xylene); (**E’**) POE-*g*-MA_IM_ (PP partially etched after 3 h in hot xylene).

The dispersion and distribution of the PET fibrils were examined on different scales of magnification using both POM (Figure 3, left column) and SEM (Figure 3, right column). Figure 3A represents a micrograph of a non-compatibilized MFC sample. Various dark regions can be found along the analysed sample, which are in fact clusters of PET fibrils. As the matching SEM picture (Figure 3A’) confirms, the dispersion and distribution of the PET phase are not very adequate. It is known that during drawing the coalescence effect causes the formation of very long microfibrils. However, as they have high aspect ratios, they may break during the injection moulding under high shear rate and may therefore stick together, thus forming fibril clusters [5]. Although it is difficult to determine the length of the PET microfibrils, they are assumed to be quite long, with an average diameter of 0.60 µm (Table 2).

Similarly, the POE_EXT_ and POE_IM_ samples are depicted in Figure 3B,D, respectively. Both POM and SEM micrographs again show a non-uniform distribution of the microfibrils and some fibril bundles along the analysed sample surfaces. Table 2 indicates that the fibril diameter of POE_EXT_ was higher than in other samples (i.e., 0.80 µm, compared to 0.70 µm in POE_IM_). In POE_IM_, the long fibrils made during the cold drawing were preserved during injection moulding. It is quite clear that non-functionalized rubber will not have a significant effect, whether it is added during extrusion or injection moulding, because it will only act as a third phase due to non-existent functional group.

On the other hand, POE-*g*-MA_EXT_ displays morphologies with both good dispersion and distribution of the PET microfibrils (Figure 3C,C’). The microfibrils appear much shorter compared to the other samples and the average fibril diameter was found to be lower (0.50 µm). In this case, the addition of compatibilizer first prevents coalescence during blending, thus reducing the starting diameter of the PET spheres and therefore also the length and diameter of PET fibrils in the MFC [15].

The POE-*g*-MA_IM_ sample is represented in Figure 3E. Although this sample shows morphology with poorly distributed fibrils, the fibrils appear to be quite long, with an average diameter of 0.60 µm. It was expected to preserve the long microfibrils made during cold drawing, as the compatibilizer was added in the injection moulding step. Fakirov et al. [15] stated that, if that was the case, the high aspect ratio of the fibrils would not be reduced. However, their distribution is not as effective as expected. This could be due to the PET fibrils being in a solid state during injection moulding, which hinders both the migration of POE-*g*-MA to the interface and the reactivity of the MA group towards the end hydroxyl groups.

### 3.2. Development of Crystalline Morphologies

**Figure 4:**
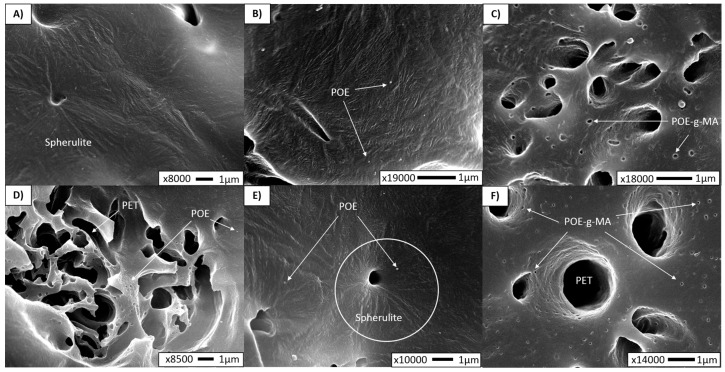
SEM micrographs of cryogenically fractured surface under liquid nitrogen of the injection moulded samples: (**A**) MFC (spherulite around fibril hole); (**B**) POE_EXT_ (randomly oriented lamellae); (**C**) POE-*g*-MA_EXT_ (non-reacted compatibilizer particles dispersed into matrix); (**D**) POE_IM_ (rubber located at the interface); (**E**) POE_IM_ (spherulite orientation around the fibril hole); and (**F**) POE-*g*-MA_IM_ (compatibilizer particles located at the interface).

Additional high-magnification SEM experiments were carried out to investigate the location of the additives and the influence of the PET fibrils rubber and compatibilizer on the PP crystalline structure. Although SEM is not the preferred method to visualize the spherulitic structure, we could observe some crystalline structures under high magnifications. The average spherulite size was measured with SALS (Supporting Information, Figure S1), and the resulting diameters are listed in Table 2. It can be noted that PP spherulite size is drastically lowered in all MFCs compared to the neat PP sample, making the detection via SEM more difficult. However, in Figure 4, showing high magnification micrographs, the spherulite structure can be detected around the hole of the etched PET fibril in the MFC sample (Figure 4A). The average spherulite diameter in the MFC was found to be 9 µm, which is roughly 60% lower than the crystal size in neat PP. This would indicate that α-crystals are present in the composite. In addition to this, XRD measurements have confirmed the presence of PP α-spherulites in all samples, as the planes (110), (040), (130), (111) and (041) were observed at 2θ = 14.1°, 17.1°, 18.6°, 21.3° and 22°, respectively (Supporting information, Figure S2). These are the typical reflections of the α-crystals [26,27].

Furthermore, POE_EXT_ and POE-*g*-MA_EXT_ show the lowest spherulitic radius of 5.5 and 4.2 µm, while POE_IM_ and POE-*g*-MA_IM_ exhibit diameters of 7.2 and 7.6 µm, respectively. POE-*g*-MA_EXT_ (Figure 4C) possesses well dispersed and distributed PET fibres which will have a strong nucleating effect on the PP matrix, regardless of whether they are covered by compatibilizer. The functionalized compatibilizer will be more prone to migrate towards the interface than the rubber. This is evidenced in Figure 4D (POE_IM_) and 4F (POE-*g*-MA_IM_), which indicate the difference in location of the rubber versus the compatibilizer.

Besides POE-*g*-MA at the interface, non-reacted compatibilizer particles were found in the PP matrix as well (Figure 4C,F). As there is always some amount of the compatibilizer that will not react with the PET during melt blending, this amount is dispersed through the matrix and between the microfibrils. These isolated POE-*g*-MA particles may also act as nucleation sites for PP [28], which explains why the nucleating effect is the most pronounced and the lowest PP crystal size is achieved. Additionally, in POE_IM_, a spherulitic orientation is observed around the fibril hole (Figure 4E), which confirms the nucleating effect of the PET.

As far as the SEM observations are concerned, it is challenging to discuss the orientation of the lamellae. It seems that the random orientation of lamellae exists in the sample POE_EXT_ represented in micrograph 4B. Moreover, as Friedrich et al. [29] explained, the organization of lamellae depends on how close the crystallites are to the surface of the microfibril. Far away from the fibril, in the bulk polymer, the lamellae are randomly dispersed with no preferred direction of orientation, which could confirm our previous statement. However, various research studies [21,29,30] conducted within the same or similar compositions (PP/PET, LDPE/PET) have shown the lamellae orientation in a normal direction to the fibril.

### 3.3. Crystallinity Development

To study the melting and crystallization behaviour of the composites, the samples were analysed via differential scanning calorimetry (DSC). Table 3 lists the melting temperature (*T*_m_), peak crystallization temperature (*T*_c_), the temperature at the beginning and end of crystallization during cooling (*T*_c_^onset^ and *T*_c_^endset^), and the calculated percentage of crystallinity (α_c_). These results are considered along those already presented in Table 2 (i.e., the PP spherulitic sizes and the PET fibril diameters). Statistically, there is a significant difference between the *T*_m_ of neat PP and all MFC-based materials, which in turn do not significantly differ from one another. Due to relatively high variations, there are no significant differences in α_c_ between all reported materials. Apparent differences in means will be discussed, however.

All materials show a single melting peak of PP, thus confirming the continued presence of α-crystalline modification, as detected via XRD.

The average *T*_c_ of neat PP is found at 118.8 °C but in pure MFC, POE_EXT_ and POE_IM_ is increased and amounts to approximately 123 °C. 

As has already been observed in the study of crystalline morphology, long PET microfibrils will act as nucleating agents for the PP matrix. This potentially results in imperfect growth of the PP crystals, which become smaller and more numerous [21,31,32]. In this case, the presence of long microfibrils in MFC enables the crystallization to start roughly 5 °C earlier than in neat PP. The total crystalline fraction is unaffected here, but indeed spherulite sizes are severely reduced, from around 22 to around 9 µm.

A similar trend can be observed in POE_EXT_ and POE_IM_ concerning the onset of crystallization, indicating that the nucleating function of the PET fibres remains uninhibited. As POE contains no structural elements that could interact with PET, it is considered to be dispersed within the PP matrix, thus not affecting the PET fibre shape or the PP-PET interface. However, there is a noticeable effect on the crystallinity of the PP matrix. It is well known that POE-type polymers will act as a nucleating agent for the a-crystals of PP [28,33,34]. Moreover, Danesi et al. [35] demonstrated many years ago that a secondary POE phase will be finely and uniformly dispersed if the viscosity of the POE is significantly lower than that of the PP matrix, as is the case here. This was confirmed in the SEM images above, which show fine droplets of POE (Figure 4D). Since POE_EXT_ benefits from already having POE present in a twin screw compounding step, it stands to reason that the dispersion of the rubber will be markedly better for POE_EXT_ than for POE_IM_. It is this increased dispersion of the rubber throughout the matrix that is responsible for the smaller crystallite sizes of PP for POE_EXT_. Average crystallinity appears to be higher for both POE materials, compared to binary MFC. This is not only due to the nucleating effect of the dispersed rubber but, as postulated by Martuscelli et al. [34], POE might also selectively extract from the PP more defective polymer chains into its amorphous phase, thus leaving a more stereoregular PP behind and increasing crystallinity.

Next, the composites with compatibilizer will be considered, for POE-*g*-MA_EXT_ crystallization is once more shifted to the level of neat PP. *T*_c_^onset^ and *T*_c_^endset^ were observed for all samples. For POE-*g*-MA_EXT_, PP crystallization started later at 121.5 and finished at 109.3 °C, which implies that it also crystalizes faster than other samples. Crystallite sizes are the lowest for this material, while overall crystallinity remains in the higher levels. It is our understanding that, given the affinity of the MA group to PET groups, the compatibilizer at least partially migrates towards the PP-PET interface during the compounding (EXT) step and there reacts with the PET. This has several effects: (i) during MFC production, coalescence of PET is inhibited, leading to shorter fibres (as was shown in SEM image 3C); and (ii) the compatibilizer will to some degree cover the PET fibres with regard to the PP matrix, thus inhibiting the nucleating effect of PET for the PP matrix. A nucleation resulting in a high α_c_ still occurs via the compatibilizer phase, but this will not affect the crystallization onset of PP as the PET fibres did: PET fibres are already in solid phase at the moment of potential PP nucleation, whereas the POE backbone is mostly amorphous and the small amount of crystallization that could occur, does so at much lower temperatures. This was confirmed in DSC analysis of the POE and POE-*g*-MA component (Supporting information, Figure S3). Some of the POE-*g*-MA is assumed to be dispersed throughout the matrix as well, given the high α_c_ and the very homogeneous structures observed in POM (Figure 4C).

In the case of POE-*g*-MA_IM_, the same significant decrease in *T*_c_ (compared to the MFC and POE materials) was noticed, indicating that some of the compatibilizer does migrate to the PP-PET interface, even when added during injection moulding. It is remarkable that injection moulding temperatures are much lower than compounding temperatures. The PET is in solid state, which hinders both the migration of POE-*g*-MA to the interface and lowers the reactivity of the MA groups towards the end hydroxyl groups. PET fibres remain relatively long, as there is no compatibilizer yet to inhibit their coalescence during drawing.

It was observed in the morphology study (Figure 4F) that the compatibilizer was located in both the matrix and at the interface. As a result, here as well, the POE backbones can provide matrix-wide nucleation of the PP. However, as with the difference between POE_EXT_ and POE_IM_, the dispersion of POE-*g*-MA is less efficient when the compounding step is missing, leading to spherulite sizes of the same order as POE_IM_. Logically, the effect of a seemingly faster crystallization as with POE-*g*-MA_EXT_ is not noted here.

## 4. Mechanical Properties

### 4.1. Tensile Behaviour

**Figure 5:**
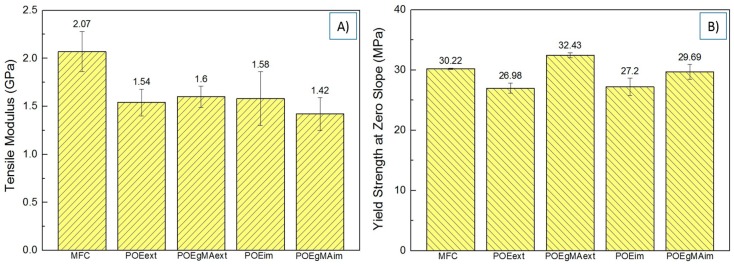
Tensile properties of MFC composites (**A**) Tensile modulus; (**B**) Yield strength at zero slope.

The tensile modulus and yield strength of the composites are given in Figure 5. Strain-at-break ε_b_ and strain-at-yield ε_y_ are reported in Table 4.

**Figure 6:**
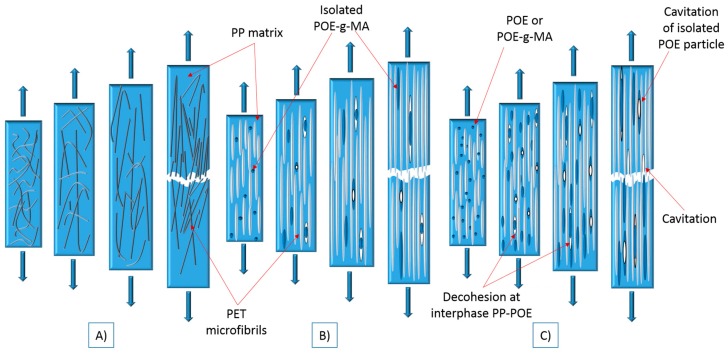
Tensile fracture model: (**A**) Case MFC; (**B**) POE-*g*-MA_EXT_; (**C**) Case POE_EXT_, POE_IM_ or POE-*g*-MA_IM_.

The bar charts indicate that MFC obtains a relatively high modulus. This could be explained by the extensive load-bearing capacity of the long PET fibrils. The interfacial area between microfibrils and matrix is large enough and some interfacial contact is assumed to exist, even without the presence of a compatibilizer. During the elongation, the matrix is expected to exert pressure on the fibrils, thus producing a high frictional force and preventing the composite from deforming. This results in constrained cavitation formation and very small ultimate elongation (Figure 6A) [36], as confirmed by the low ε_b_.

Among all other composites containing POE and POE-*g*-MA added during extrusion or injection moulding, no significant differences are found for the modulus. These composites all obtain a lower stiffness due to the presence of the rubber and the compatibilizer with a soft backbone.

A significant increase was found in the yield strength of POE-*g*-MA_EXT_ compared to non-compatibilized MFC, POE_EXT_, POE_IM_ and POE-*g*-MA_IM_ (*p* < 0.001). The reason for this lies in the lower interfacial tension between the POE-*g*-MA and PET achieved during extrusion, which enhances the interfacial adhesion of PP and PET in the final composite. Figure 6B represents a tensile fracture model which can be applied to a compatibilized composite such as POE-*g*-MA_EXT_. In this model, stress transfer between matrix and fibre is excellent: both strain together while the PET fibres do most of the load bearing, until they finally fail together. Such deformation behaviour is only possible with great adhesion between the phases, which in this case is demonstrated by the high ε_y_ and ε_b_ values.

As a result, while neat MFC—being the only composite not containing some rubber fraction—obtains the highest stiffness, POE-*g*-MA_EXT_ surpasses it in terms of strength and strain behaviour due to the presence of the compatibilizer, even if the aspect ratio of the PET fibrils is reduced and the composite contains a rubber phase. This corroborates previous experimental results for in situ compatibilized MFC by Yi et al. [6].

It can also be noted that the yield strength for POE_EXT_, POE_IM_ and POE-*g*-MA_IM_ is lower due to elastomeric chains in the polymer backbone, as mentioned earlier. No differences were found between POE_EXT_ and POE_IM_ (*p* = 0.772), while there were significant differences when POE-*g*-MA_IM_ was compared to POE_EXT_ (*p* = 0.003) and POE_IM_ (*p* = 0.017). The somewhat higher yield stress for POE-*g*-MA_IM_ can be attributed to a limited interaction between the compatibilizer and PET, as proposed in Section 3.3.

G’Sell et al. [37] explained that several mechanisms could contribute to the general deformation of an MFC sample including elastomers under tension: interface decohesion, cavitation at the PP/PET interface, and the cavitation in isolated POE-*g*-MA particles. These are summarized in the fracture model for the MFCs made with POE_EXT_, POE_IM_ or POE-*g*-MA_IM_, as shown in Figure 6C.

In these composites, mostly isolated POE or POE-*g*-MA particles were found during morphology study. As observed earlier, non-functionalized POE cannot react with PET and thus a three-phase microstructure was created. The same effect was largely present in POE-*g*-MA_IM_. Under tensile test, the strain rate is low and when critical stress is reached, the rubber takes the shape of fibril. With further stretching, the rubber fibrils continuously transfer stress to the matrix and they become elongated when their yield strength is exceeded. Rubber fibril structures will be preserved during the whole fracture process [38]. In all three cases, during continuous stretching there is a substantial risk of decohesion to appear at the interphases between PET and PP, as well as POE particles and PP matrix and the cavitation in isolated POE particle [37]. Strain levels are improved by the addition of the elastomer, but effective decohesion between the PP matrix and the PET fibres is likely, given the much larger ε_y_ and ε_b_ demonstrated by POE-*g*-MA_EXT_.

G’Sell et al. [37] reported that in the case of decohesion of the matrix from a nodule or cavitation in the rubber particle (Figure 6C) by stretching the matrix, large voids can occur on poles of the rubber. These voids play a significant role at the end of deformation because their presence make a composite fracture easier, causing a decrease in modulus and strength.

### 4.2. Impact Behaviour

Numerous studies have explored how a high fracture toughness may be achieved, for example by using intrinsically tough matrices or rubber modified or by incorporating different fibres as reinforcements [1,2,3,4,37,39,40]. Similarly, the impact behaviour of semicrystalline polymers has been studied extensively, as well as how this may be improved [17,18,39,41,42,43].

**Figure 7:**
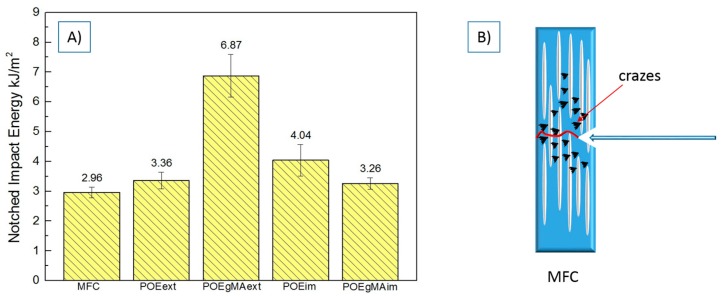
(**A**) Comparison of impact strength of MFC composites; (**B**) Impact fracture model of MFC.

Comparing the toughness of the composites (Figure 7A), we observed that the POE-*g*-MA_EXT_ gave the highest value for the impact energy. This could be explained by the fact that there is high interfacial adhesion between the fibrils and matrix in POE-*g*-MA_EXT_, as polar carboxyl groups of MA grafted onto POE backbone improve the adhesion with the PET phase. In addition to the interfacial adhesion between the oriented PET fibrils and the PP matrix, as has been mentioned earlier, both the size and amount of PP crystallites play an important role in determining impact strength. The PP spherulites become more imperfect and smaller due to the presence of PET fibrils, which may increase toughness [5,18]. As mentioned earlier, the spherulite size in POE-*g*-MA_EXT_ was the lowest, which obviously contributed to an increase in toughness.

Although the fibrils formed in this composite are shorter, they have a higher resistance by better dissipation of the impact energy. It is known that the rubber phase could initiate crazes thus contributing to the absorption of the impact energy to block the crack propagation [44]. As was reported by Perkins et al. [18], polymer toughness can be improved by optimizing the crystalline morphology, incorporating a discrete rubbery phase or by adding fibres as reinforcements to polymer matrices.

Figure 7B represents the impact fracture model of the MFC and shows that the crazing mechanism is in fact present. Crazing is one of the preferential deformation mechanisms which may prevent the further development of the craze into crack along the impact direction [44]. In the composite with good fibrils dispersion and distribution, the crack will propagate along the impact direction, but it will also deflect for an angle from the impact direction. The fibrils may induce a crack deflection perpendicular to the impact direction and transmit the stress to the matrix. This will make the matrix participate more actively in the stress transfer, which in turn will increase absorbed energy.

Compared to pure MFC, composites POE_EXT_, POE_IM_ and POE-*g*-MA_IM_ show a slight but still significant increase in impact energy. As was explained earlier, all these composites have obtained three-phase morphology, where besides the PET fibrils in the matrix, POE or POE-*g*-MA particles are also dispersed. The highest increase in impact strength was achieved in POE_IM_, and compared with POE_EXT_ (*p* = 0.035) and POE-*g*-MA_IM_ (*p* = 0.015), significant differences were found. The reason for this could be the existence of both spherical POE particles and long PET microfibrils, which both have acted as nucleating sites for PP matrix and could more effectively include the matrix in absorbing the energy. As Wang et al. [44] have explained, if the composite contains spherical rubber reinforcements, the material could be toughened only when the stress field around the rubber particles overlap and go through the matrix. No significant differences could be observed between POE_EXT_ and POE-*g*-MA_IM_ (*p* = 0.533), but compared to pure MFC (*p* = 0.030), significant differences can in fact be found. These differences may be the result from the third rubber phase, as stress transfer is not continuous and the PET microfibrils cannot reach higher levels of the energy absorption due to their poor dispersion and distribution.

### 4.3. Comparison to Non-Fibrillated Blends

In the end, by adding the compatibilizer, we have seen that POE-*g*-MA_EXT_ improved in terms of both yield and impact strength, as well as in strain at break compared to all MFCs. Not unexpectedly, this comes at a cost in terms of stiffness. Given that an increased stiffness is one of the largest gains achieved by producing MFCs rather than just using non-fibrillated blends, we found it necessary to hold our results against similar experiments for neat PP, blend or additivated undrawn blends (here referred to as IMB, Injection Moulded Blends), with the subscript denominating the additive (added in the compounding step, EXT). These results are summarized in Table 5, together with the results of MFC and POE-*g*-MA_EXT_, which have been added for clarity’s sake. The highest value achieved is marked in bold.

None of the composites, including POE-based additives, achieve the high modulus of neat MFC. IMB_POEext_ and POE-*g*-MA_EXT_ both manage to maintain a modulus of around 1600 MPa, which is close to that of the neat IMB. However, only POE-*g*-MA_EXT_ shows an additional large increase in impact strength and strain levels, as well as a small improvement in yield strength. IMB_POE-*g*-MAext_ does manage to match its impact strength, but at an unacceptable cost in stiffness.

The formulation of POE-*g*-MA_EXT_ shows that also short fibrils may act as good reinforcement for the PP matrix and that at least equal importance should be given to the stress transfer possibilities between matrix and fibre, which in this case is effectively facilitated by the compatibilizer POE-*g*-MA.

POE-*g*-MA_EXT_ is considered the best formulation for the manufacture of a PP/PET MFC with all-round good mechanical properties.

## 5. Conclusions

This paper presented a comprehensive study on the relationships between the microstructure and mechanical properties of PP/PET microfibrillar composites (MFCs). MFCs were prepared in a weight ratio 80/20 PP/PET via three-step processing with addition of 6 wt % of POE or POE-*g*-MA during extrusion or injection moulding.

Under POM microscopy, the presence of PP spherulites was observed in neat PP, and the skin-core structure was established. Both POM and SEM microscopies confirmed the non-uniform dispersion and distribution of the fibrils in the samples MFC, POE_EXT_, POE_IM_ and POE-*g*-MA_IM_, while in POE-*g*-MA_EXT_ the dispersion and distribution were found to be very good. Furthermore, we were able to examine the effect of adding POE or POE-*g*-MA in different steps of processing by means of the SEM micrographs. In the composites POE_EXT_, POE_IM_ and POE-*g*-MA_IM_ the three-phase microstructure was developed, but the long microfibrils were preserved. Shorter fibrils were obtained in POE-*g*-MA_EXT_, due to the addition of the compatibilizer during extrusion, which prevented a coalescence of the PET particles. In addition to the development of phase morphology, crystalline morphology was investigated. The MFCs without an amorphous phase were analysed via SEM under high magnification. The spherulite structure developed in most of the samples, and a random orientation of the lamellae was noted in bulk polymer, as well as some spherulite orientation around the fibril holes. PP spherulite size was drastically lower in all MFC samples, supporting the theory that PET fibrils may act as heterogeneous nucleating points for the PP matrix.

XRD measurements and melting behaviour have confirmed the formation of α-spherulites in all composites. DSC analysis, along with SALS measurements of spherulite sizes, showed that both POE and PET fibrils are good nucleators for PP, in which only PET also effects a shift in *T*_c_. Furthermore, these combined results suggest that POE will be dispersed within the matrix exclusively, while POE-*g*-MA will migrate to the PP-PET interface. It does so, even when added only during injection moulding, but the strong compatibilizing effect will only occur if it is already added during the compounding of the blend.

The mechanical results have confirmed that the microstructure and properties are significantly affected by adding the rubber and the compatibilizer in different processing steps. The impact strength of POE-*g*-MA_EXT_ was found to be superior compared to all other composites. All composites have shown a brittle breakage during the tensile tests, except POE-*g*-MA_EXT_, where necking was observed. In POE-*g*-MA_EXT_, a significant increase in yield strength and at strain at break was noted, as the compatibilizer added during extrusion caused a better interfacial adhesion between PP and PET in the final composite.

It has been demonstrated that POE-*g*-MA_EXT_ shows better all-round mechanical properties compared to both IMBs and MFCs. The PET short fibrils can act as excellent reinforcement for the PP matrix, when they are produced with an addition of rubber-based compatibilizer during extrusion. The main objective of this study was to evaluate the potential of adding the compatibilizer only in the injection moulding step, as suggested by Fakirov et al. Considering all experimental results, we can conclude that, while postponing the compatibilizer addition does conserve the long fibrils, it does not create the best mechanical properties for a compatibilized PP/PET MFC. Mixing in the compatibilizer prior to drawing does achieve this, despite the reduction in fibril length.

## Figures and Tables

**Table 1:** Formulations of the composites.

Material	PP/PET wt %	CA wt %
MFC	80/20	0
POE_EXT_	75.2/18.8	6 _EXT_
POE-*g*-MA_EXT_	75.2/18.8	6 _EXT_
POE_IM_	75.2/18.8	6 _IM_
POE-*g*-MA_IM_	75.2/18.8	6 _IM_

**Table 2:** Average diameter of PP spherulites and PET fibrils in the composites.

Material	Spherulite Size (µm)	Fibril Diameter (µm)
PP MFC	22.3 ± 0.8 8.9 ± 0.4	- 0.6 ± 0.2
POE_EXT_	5.5 ± 0.7	0.8 ± 0.3
POE-*g*-MA_EXT_	4.2 ± 0.3	0.5 ± 0.1
POE_IM_	7.2 ± 0.4	0.7 ± 0.3
POE-*g*-MA_IM_	7.6 ± 0.4	0.6 ± 0.2

**Table 3:** Thermal properties of PP, MFC, POE_EXT_, POE-*g*-MA_EXT_, POE_IM_, POE-*g*-MA_IM_ during heating and cooling.

Material	*T*_m_^PP^ (°C)	*T*_c_^PP^ (°C)	*T*_c_^onset^ (°C)	*T*_c_^endset^ (°C)	α_c_^PP^ (%)
PP MFC	171.5 ± 0.2 170.2 ± 0.8	118.8 ± 0.1 122.8 ± 0.7	122.5 ± 1.2 127.2 ± 0.2	108.1 ± 1.4 112.8 ± 0.4	47.05 ± 0.8 47.35 ± 2.2
POE_EXT_	170.1 ± 0.4	123.1 ± 0.6	127.1 ± 0.2	113.2 ± 0.1	48.40 ± 0.5
POE-*g*-MA_EXT_	170.1 ± 0.4	118.1 ± 0.6	121.5 ± 0.2	109.3 ± 0.9	50.01 ± 1.9
POE_IM_	169.3 ± 0.9	122.9 ± 0.4	127.1 ± 0.2	113.2 ± 0.3	51.65 ± 2.9
POE-*g*-MA_IM_	170.1 ± 1.2	116.4 ± 0.6	123.0 ± 0.1	108.3 ± 0.5	49.44 ± 2.8

**Table 4:** Strain at break of the composites.

Material	Strain at Yield (%)	Strain at Break (%)
MFC	3.74 ± 0.9	5.29 ± 0.9
POE_EXT_	6.84 ± 0.2	10.69 ± 1.7
POE-*g*-MA_EXT_ POE_IM_ POE-*g*-MA_IM_	11.90 ± 0.3 6.52 ± 1.1 8.91 ± 1.4	190.37 ± 162.8 9.70 ± 2.3 11.42 ± 2.4

**Table 5:** Mechanical properties of injection moulding blends.

Material	Tensile Modulus GPa	Yield Strength MPa	Strain at Break %	Impact Strength kJ/m^2^
PP IMB	1.47 ± 0.1 1.72 ± 0.1	17.3 ± 1.2 24.8 ± 0.4	>500 4.78 ± 0.5	1.45 ± 0.2 2.06 ± 0.4
IMB_POEext_	1.58 ± 0.1	27.59 ± 0.7	10.43 ± 2.6	3.04 ± 0.5
IMB_POE-g-MAext_ POE-*g*-MA_EXT_ MFC	1.21 ± 0.1 1.60 ± 0.1 2.07 ± 0.2	29.54 ± 0.1 32.43 ± 0.4 30.22 ± 0.1	10.48 ± 4.8 190.32 ± 162.8 5.29 ± 0.9	6.55 ± 1.9 6.87 ± 0.7 2.96 ± 0.2

## Supplementary Materials

The following are available online at http://www.mdpi.com/2073-4360/10/3/291/s1.

## References

[1] Fu S.-Y., Lauke B., Mäder E., Yue C.-Y., Hu X. (2000). Tensile properties of short-glass-fiber-and short-carbon-fiber-reinforced polypropylene composites. Compos. Part A Appl. Sci. Manuf..

[2] Bao S., Liang G., Tjong S. (2012). Part II: Polymer-Polymer Composites with Premade Fibrous Reinforcement: Fracture Behavior of Short Carbon Fiber Reinforced Polymer Composites. Synthetic Polymer–Polymer Composites.

[3] McCardle R., Bhattacharyya D., Fakirov S. (2012). Effect of Reinforcement Orientation on the Mechanical Properties of Microfibrillar PP/PET and PET Single-Polymer Composites. Macromol. Mater. Eng..

[4] Fakirov S. (2012). The Concept of Micro-or Nanofibrils Reinforced Polymer-Polymer Composites.

[5] Kuzmanović M., Delva L., Cardon L., Ragaert K. (2016). The Effect of Injection Molding Temperature on the Morphology and Mechanical Properties of PP/PET Blends and Microfibrillar Composites. Polymers.

[6] Yi X., Xu L., Wang Y.-L., Zhong G.-J., Ji X., Li Z.-M. (2010). Morphology and properties of isotactic polypropylene/poly(ethylene terephthalate) in situ microfibrillar reinforced blends: Influence of viscosity ratio. Eur. Polym. J..

[7] Chiu H.-T., Hsiao Y.-K. (2006). Compatibilization of poly(ethylene terephthalate)/polypropylene blends with maleic anhydride grafted polyethylene-octene elastomer. J. Polym. Res..

[8] Moini Jazani O., Khoramabadi M.A., Salehi M.M., Riazi H., Soltanokottabi F. (2015). Effective parameters on the phase morphology and mechanical properties of PP/PET/SEBS ternary polymer blends. J. Part. Sci. Technol..

[9] Jayanarayanan K., Thomas S., Joseph K. (2015). Effect of compatibilizer on the morphology development, static and dynamic mechanical properties of polymer-polymer composites from LDPE and PET. Int. J. Plast. Technol..

[10] Heino M., Kirjava J., Hietaoja P. (1997). Compatibilization of polyethylene terephthalate/polypropylene blends with styrene–ethylene/butylene–styrene (SEBS) block copolymers. J. Appl. Polym. Sci..

[11] Asgari M., Masoomi M. (2012). Thermal and impact study of PP/PET fibre composites compatibilized with glycidyl methacrylate and maleic anhydride. Compos. Part B Eng..

[12] Zhang X., Li B., Wang K., Zhang Q., Fu Q. (2009). The effect of interfacial adhesion on the impact strength of immiscible PP/PETG blends compatibilized with triblock copolymers. Polymer.

[13] Pang Y., Jia D., Hu H., Hourston D., Song M. (2000). Effects of a compatibilizing agent on the morphology, interface and mechanical behaviour of polypropylene/poly(ethylene terephthalate) blends. Polymer.

[14] Entezam M., Khonakdar H.A., Yousefi A.A., Jafari S.H., Wagenknecht U., Heinrich G., Kretzschmar B. (2012). Influence of interfacial activity and micelle formation on rheological behavior and microstructure of reactively compatibilized PP/PET blends. Macromol. Mater. Eng..

[15] Fakirov S., Bhattacharyya D., Lin R., Fuchs C., Friedrich K. (2007). Contribution of coalescence to microfibril formation in polymer blends during cold drawing. J. Macromol. Sci. Part B Phys..

[16] Friedrich K., Evstatiev M., Fakirov S., Evstatiev O., Ishii M., Harrass M. (2005). Microfibrillar reinforced composites from PET/PP blends: Processing, morphology and mechanical properties. Compos. Sci. Technol..

[17] Schrauwen B.B. (2003). Deformation and Failure of Semi-Crystalline Polymers: Influence of Micro and Molecular Structure. Ph.D. Thesis.

[18] Perkins W.G. (1999). Polymer toughness and impact resistance. Polym. Eng. Sci..

[19] Zhao Z., Yang Q., Kong M., Tang D., Chen Q., Liu Y., Lou F., Huang Y., Liao X. (2015). Unusual hierarchical structures of micro-injection molded isotactic polypropylene in presence of an in situ microfibrillar network and a β-nucleating agent. RSC Adv..

[20] Harel H., Marom G. (1998). On crystalline interfaces in composite materials. Acta Polym..

[21] Quan H., Li Z.-M., Yang M.-B., Huang R. (2005). On transcrystallinity in semi-crystalline polymer composites. Compos. Sci. Technol..

[22] Yalcin B., Cakmak M. (2004). Superstructural hierarchy developed in coupled high shear/high thermal gradient conditions of injection molding in nylon 6 nanocomposites. Polymer.

[23] Meeten G. (1986). Refraction and extinction of polymers. Optical Properties of Polymers.

[24] Jayanarayanan K., Bhagawan S., Thomas S., Joseph K. (2008). Morphology development and non isothermal crystallization behaviour of drawn blends and microfibrillar composites from PP and PET. Polym. Bull..

[25] Xu J., Ye H., Zhang S., Guo B. (2017). Organization of Twisting Lamellar Crystals in Birefringent Banded Polymer Spherulites: A Mini-Review. Crystals.

[26] Zhang S., Lin W., Zhu L., Wong C.P., Bucknall D.G. (2010). γ-Form Transcrystals of Poly propylene) Induced by Individual Carbon Nanotubes. Macromol. Chem. Phys..

[27] Jayanarayanan K., Thomas S., Joseph K. (2011). In situ microfibrillar blends and composites of polypropylene and poly(ethylene terephthalate): Morphology and thermal properties. J. Polym. Res..

[28] Marcinčin A. (2002). Modification of fiber-forming polymers by additives. Prog. Polym. Sci..

[29] Friedrich K., Ueda E., Kamo H., Evstatiev M., Krasteva B., Fakirov S. (2002). Direct electron microscopic observation of transcrystalline layers in microfibrillar reinforced polymer-polymer composites. J. Mater. Sci..

[30] Li Z.M., Li L.B., Shen K.Z., Yang W., Huang R., Yang M.B. (2004). Transcrystalline morphology of an in situ microfibrillar poly(ethylene terephthalate)/poly(propylene) blend fabricated through a slit extrusion hot stretching-quenching process. Macromol. Commun..

[31] Denchev Z., Dencheva N. (2012). Preparation, mechanical properties and structural characterization of microfibrillar composites based on polyethylene/polyamide blends. Synthetic Polymer-Polymer Composites.

[32] Xu L., Zhong G.-J., Ji X., Li Z.-M. (2011). Crystallization behavior and morphology of one-step reaction compatibilized microfibrillar reinforced isotactic polypropylene/poly(ethylene terephthalate)(iPP/PET) blends. Chin. J. Polym. Sci..

[33] Karger-Kocsis J., Kallo A., Szafner A., Bodor G., Senyei Z. (1979). Morphological study on the effect of elastomeric impact modifiers in polypropylene systems. Polymer.

[34] Martuscelli E., Silvestre C., Abate G. (1982). Morphology, crystallization and melting behaviour of films of isotactic polypropylene blended with ethylene-propylene copolymers and polyisobutylene. Polymer.

[35] Danesi S., Porter R.S. (1978). Blends of isotactic polypropylene and ethylene-propylene rubbers: Rheology, morphology and mechanics. Polymer.

[36] Chen Y., Zhong G., Li Z. (2012). Microfibril Reinforced Polymer-Polymer Composites via Hot Stretching: Preparation, Structure and Properties.

[37] G’Sell C., Bai S.-L., Hiver J.-M. (2004). Polypropylene/polyamide 6/polyethylene–octene elastomer blends. Part 2: Volume dilatation during plastic deformation under uniaxial tension. Polymer.

[38] Shi D., Liu E., Tan T., Shi H., Jiang T., Yang Y., Luan S., Yin J., Mai Y.-W., Li R.K. (2013). Core/shell rubber toughened polyamide 6: An effective way to get good balance between toughness and yield strength. RSC Adv..

[39] Argon A., Cohen R. (2003). Toughenability of polymers. Polymer.

[40] Evstatiev M., Fakirov S., Bechtold G., Friedrich K. (2000). Structure-property relationships of injection-and compression-molded microfibrillar-reinforced PET/PA-6 composites. Adv. Polym. Technol..

[41] Redakcji O. (2014). Plastic deformation and cavitation in semicrystalline polymers studied by X-ray methods. Polimery.

[42] Meijer H.E., Govaert L.E. (2005). Mechanical performance of polymer systems: The relation between structure and properties. Prog. Polym. Sci..

[43] Galeski A. (2003). Strength and toughness of crystalline polymer systems. Prog. Polym. Sci..

[44] Wang J., Wu H., Guo S. (2016). Realizing simultaneous reinforcement and toughening in polypropylene based on polypropylene/elastomer via control of the crystalline structure and dispersed phase morphology. RSC Adv..

