# Supplementary material for: Development of Crystalline Morphology and Its Relationship with Mechanical Properties of PP/PET Microfibrillar Composites Containing POE and POE-g-MA
Source: Polymers (Basel). 2018 Mar 8;10(3):291. doi: 10.3390/polym10030291 (PMC6414988; doi:10.3390/polym10030291)
Supplement: Supplementary file 1 [file polymers-10-00291-s001.pdf]

Supporting information

2.1. Materials – datasheets

PP 575P

Product Description
PP 575P is specially developed for producing rigid injection molding articles for general purpose applications. It gives consistent processability and high gloss in the products.

Typical Applications
PP 575P can be used mainly for houseware articles, caps, closures, containers and toys.

Typical data

Properties	Unit	Value (1)	ASTM Method
**Resin Properties**			
Melt Flow Rate @ 230°C & 2.16 kg load	g/10 min.	11	D 1238
Density @ 23°C	kg/m^3	905	D 792
**Mechanical Properties (2)**			
Tensile Strength @ Yield	MPa	35	D 638
Tensile Elongation @ Yield	%	11	D 638
Flexural Modulus (1% Secant)	MPa	1600	D 790A
Notched Izod Impact Strength @ 23°C	J/m	22	D 256
Rockwell Hardness, R-Scale	-	104	D 785
**Thermal Properties(2)**			
Vicat Softening Point	°C	153	D 1525B
Heat Deflection Temperature @ 455 KPa	°C	98	D 648

(1) Typical values; not to be construed as specification limits.
(2) Based on injection molded specimens.

Processing Conditions
Barrel temperature range: 200 - 225°C
Mold Shrinkage: 1.2 - 2.5% depending on wall thickness and processing conditions
Mold Temperature: Normally 15 - 40°C, upto 65°C for thick parts

Food Regulation
PP 575P is suitable for Food contact application. Detailed information is provided in relevant Material Safety Datasheet and for additional specific information please contact SABIC local representative for certificate.

Storage and Handling
PP resin should be stored to prevent a direct exposure to sunlight and/or heat. The storage area should also be dry and preferably don’t exceed 50°C. SABIC would not give warranty to bad storage conditions which may lead to quality deterioration such as color change, bad smell and inadequate product performance. It is advisable to process PP resin within 6 months after delivery.

LIGHTER C93

Polyethylene Terephthalate (PET) Resins

LIGHTERTM resins are polyethylene terephthalate produced from PTA and MEG. These polymers are specifically designed for the production of beverage, food and other liquid containers and for thermoforming. There are several LIGHTER PET polymers, designed for the specific performance requirements of different applications, including very good mechanical properties, excellent clarity and a wide processing range on all injection and stretch blow molding machines.

LIGHTER C93 is suitable for the production of both mineral water and carbonated soft drinks bottles.

LIGHTER C93 is recommended also for the production of extruded thermoformable sheets.

LIGHTER C93, when used unmodified and processed under good manufacturing practices, should allow packaging article production in compliance with the laws and regulations for articles in contact with foodstuffs in force in the European Union and in the United States of America. Please contact your nearest Equipolymers office regarding food contact compliance statements. The purchaser remains responsible for determining whether the use complies with all relevant regulations.

SALES SPECIFICATIONS	Unit	Method	LIGHTER C93
Intrinsic Viscosity	dl/g	1/MA/1/002	0.80 ± 0.02
Acetaldehyde	ppm	1/MA/1/004	1 max.
Color, coordinate b		1/MA/1/003	1.5 max.
Fine Particles Content	ppm	1/MA/1/001	500 max.
Moisture	%	1/MA/1/005	0.4 max.

TYPICAL VALUES	Unit	Method	LIGHTER C93
Bulk density	kg/m3	1/MA/1/008	880
Glass Transition Temperature	°C	1/MA/1/007	78
Melting Point (peak)	°C	1/MA/1/007	247
Crystallinity	%	1/MA/1/018	50 min
Weight of 100 Granules	g	1/MA/1/015	1.5
ISO Viscosity Number	ml/g	ISO 1628/5	93

Product Datasheet

ExxonMobil

Vistamaxx™ 6102

Performance Polymer

Product Description	Key Features
Vistamaxx 6102 is primarily composed of isotactic propylene repeat units with random ethylene distribution, and is produced using ExxonMobil's proprietary metallocene catalyst technology. It has excellent elastomeric properties, is easy to process and is compatible with a wide variety of materials. It is particularly good for thermoplastic and polyolefinic blends where a balance of flexibility, transparency and impact performance is required.	• Suitable for a wide range of film and compounding applications.<br>• Other typical applications include calendered or extruded profiles, foamed or blown molded goods and thermoformed products.<br>• Excellent adhesion to conventional or metallocene PP and PE.<br>• Very good elasticity, toughness and melt strength.<br>• Very low seal initiation temperature combined with high seal strength when used as sealing layer of co-extruded structures.<br>• Very good chemical resistance and long term aging.<br>• RoHS compliant.

General			
Availability [1]	• Africa & Middle East<br>• Asia Pacific	• Europe<br>• Latin America	• North America
Applications	• Blown Film<br>• Blown Molded Goods<br>• Calendered Profiles	• Cast Film<br>• Extruded Profiles<br>• Foamed Goods	• PP/TPE Modification
Uses	• Compounding	• Film	• Packaging
RoHS Compliance	• RoHS Compliant		
Form(s)	• Pellets		
Revision Date	• 01/01/2017		

Physical	Typical Value	(English)	Typical Value	(SI)	Test Based On
Density [2]	0.862	g/cm³	0.862	g/cm³	ASTM D1505
Melt Index [2] (190°C/2.16 kg)	1.4	g/10 min	1.4	g/10 min	ASTM D1238
Melt Mass-Flow Rate (MFR) [2] (230°C/2.16 kg)	3	g/10 min	3	g/10 min	ExxonMobil Method
Ethylene Content	16	wt%	16	wt%	ExxonMobil Method

Hardness	Typical Value	(English)	Typical Value	(SI)	Test Based On
Durometer Hardness (Shore A)	67		67		ASTM D2240

Mechanical	Typical Value	(English)	Typical Value	(SI)	Test Based On
Tensile Stress at 100%	324	psi	2.23	MPa	ASTM D638
Tensile Stress at 300%	402	psi	2.77	MPa	ASTM D638
Tensile Strength at Break	> 1100	psi	> 7.58	MPa	ASTM D638
Tensile Set	12	%	12	%	ExxonMobil Method
Elongation at Break	> 800	%	> 800	%	ASTM D638
Flexural Modulus - 1% Secant	2090	psi	14.4	MPa	ASTM D790

Elastomers	Typical Value	(English)	Typical Value	(SI)	Test Based On
Tear Strength (Die C)	190	lbf/in	33.3	kN/m	ASTM D624

Thermal	Typical Value	(English)	Typical Value	(SI)	Test Based On
Vicat Softening Temperature	129	°F	53.9	°C	ExxonMobil Method

ExxonMobil

Vistamaxx™ 6102
Performance Polymer

Additional Information

For data specific to chemical resistance, refer to the Technical Literature (TL), Chemical Resistance of Vistamaxx Performance Polymer.

Please contact Customer Service for food law compliance information.

Legal Statement

This product, including the product name, shall not be used or tested in any medical application without the prior written acknowledgement of ExxonMobil Chemical as to the intended use. For detailed Product Stewardship information, please contact Customer Service.

Processing Statement

Vistamaxx polymers have a wide temperature processing window. A good starting point for temperatures is 10°C above the highest melting point. This material does not require drying and can be compounded or used in a dry blend. Use conventional processing knowledge to ensure mixing of the materials.

Notes

Typical properties: these are not to be construed as specifications.

[1] Product may not be available in one or more countries in the identified Availability regions. Please contact your Sales Representative for complete Country Availability.

[2] Property specified in conventional unit of measure.

For additional technical, sales and order assistance: www.exxonmobilchemical.com/ContactUs

©2018 ExxonMobil. ExxonMobil, the ExxonMobil logo, the interlocking "X" device and other product or service names used herein are trademarks of ExxonMobil, unless indicated otherwise. This document may not be distributed, displayed, copied or altered without ExxonMobil's prior written authorization. To the extent ExxonMobil authorizes distributing, displaying and/or copying of this document, the user may do so only if the document is unaltered and complete, including all of its headers, footers, disclaimers and other information. You may not copy this document to or reproduce it in whole or in part on a website. ExxonMobil does not guarantee the typical (or other) values. Any data included herein is based upon analysis of representative samples and not the actual product shipped. The information in this document relates only to the named product or materials when not in combination with any other product or materials. We based the information on data believed to be reliable on the date compiled, but we do not represent, warrant, or otherwise guarantee, expressly or impliedly, the merchantability, fitness for a particular purpose, freedom from patent infringement, suitability, accuracy, reliability, or completeness of this information or the products, materials or processes described. The user is solely responsible for all determinations regarding any use of material or product and any process in its territories of interest. We expressly disclaim liability for any loss, damage or injury directly or indirectly suffered or incurred as a result of or related to anyone using or relying on any of the information in this document. This document is not an endorsement of any non-ExxonMobil product or process, and we expressly disclaim any contrary implication. The terms "we," "our," "ExxonMobil Chemical" and "ExxonMobil" are each used for convenience, and may include any one or more of ExxonMobil Chemical Company, Exxon Mobil Corporation, or any affiliate either directly or indirectly stewarded.

Acti-Tech® 16MA13

Product description

This Acti-Tech grade is a maleated propylene copolymer that can be used for the following applications:

- Soft compatibilizer for polar/unpolar polymers alloys
- Impact modifier of stiff polar engineering plastics
- Coupling agent for mineral HFFR fillers
- Coupling agent for WPC or glass-fiber reinforced PP

Due to the chemical nature of its backbone, Acti-Tech xxMAyy is compatible with most of polyolefins, i.e. PE, PP, EVA, EBA or POE.

Physical	Typical Value (SI)	Test
Density	0.86 g/cm^3	ISO 1183
Maleic Anhydride content	1.3%	FTIR
MVR (190°C/2.16kg)	8.5 cc/10 mn	

Processing Information

This material can be processed on any conventional extruder. Typical addition level is 5 to 15%.
This material does not require drying prior to processing.

Contact information

Nordic Grafting Company A/S
Søholm Park 1
DK 2900 Hellerup

Telephone: +45 35 43 88 43
Telefax: +45 35 43 99 43

3.2. Development of crystalline morphologies

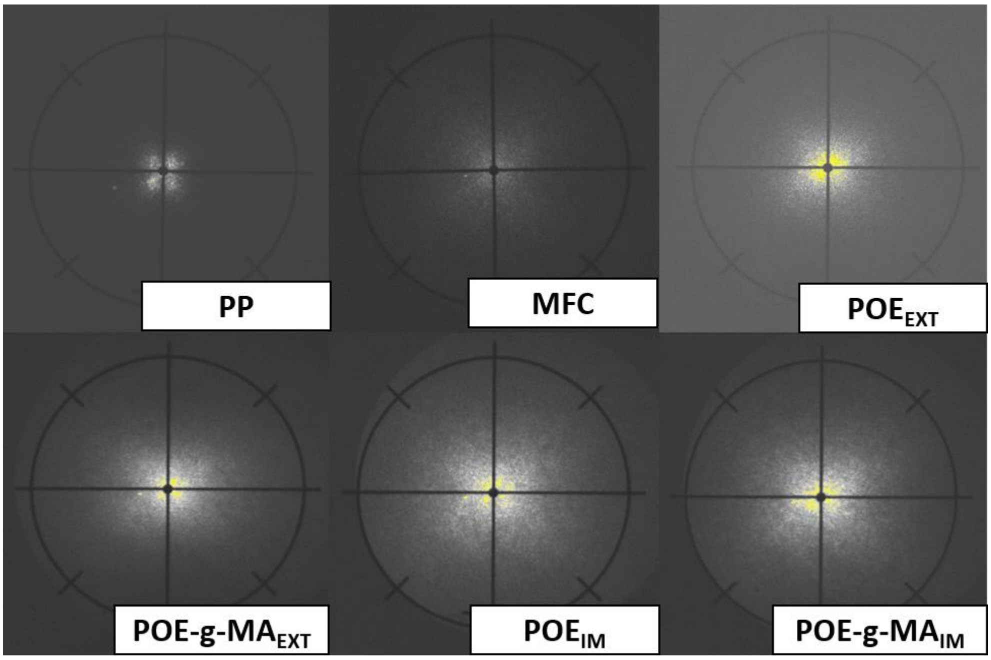

Figure S1. SALS patterns of the samples.

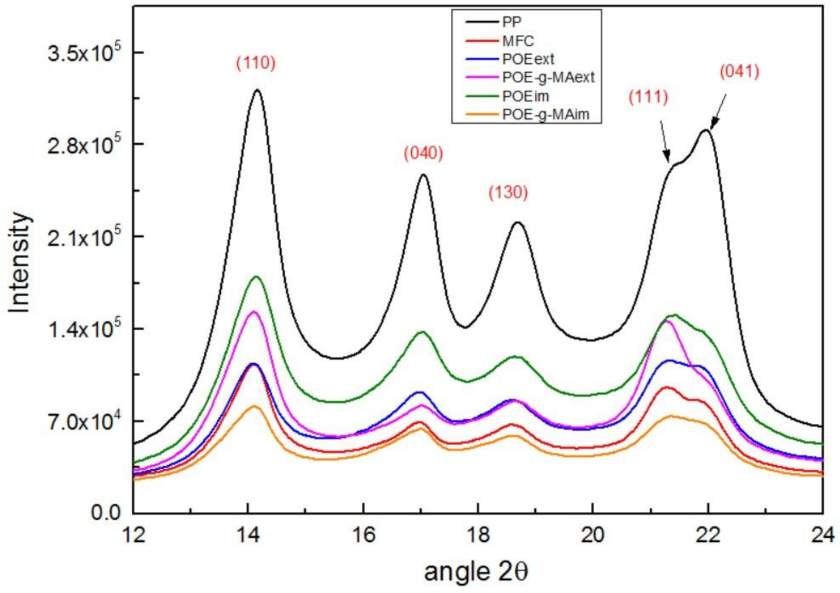

Figure S2. XRD spectra of the samples.

3.3.Crystallinity development

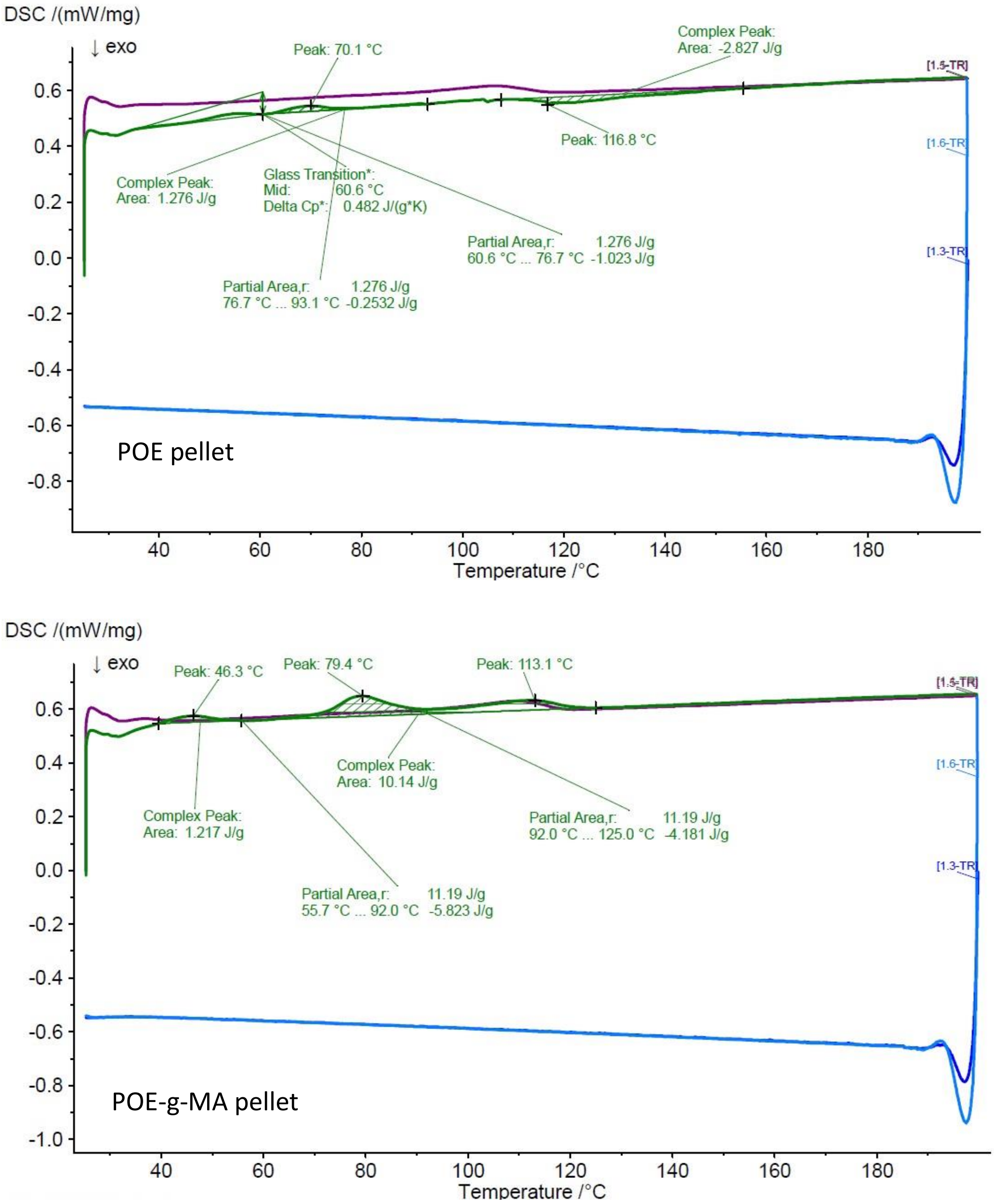

Figure S3. DSC graphs of POE and POE-g-MA pellets
